# Parental Self‐Efficacy and Family Support in Oral Health and Nutrition of Children With Disabilities

**DOI:** 10.1111/cch.70322

**Published:** 2026-08-05

**Authors:** Serdal Deniz, Derya Evgin, Rıdvan Karabulut

**Affiliations:** ^1^ Child Development Department Kayseri University Health Science Faculty Kayseri Turkey

**Keywords:** caregiving difficulty, children with disabilities, family nutrition, family support, oral health, parental self‐efficacy

## Abstract

**Background:**

Parents of children with disabilities often manage complex nutritional and oral health needs, yet the factors supporting their competence remain insufficiently understood.

**Aim:**

This study examined the relationships of parental practices, parental self‐efficacy, family support and caregiving characteristics with nutrition and oral health management among children with disabilities.

**Methods:**

A cross‐sectional, descriptive, correlational design was used. The sample comprised 195 parents of children with disabilities. Data were analysed using independent‐samples *t* tests, one‐way analysis of variance with Bonferroni post hoc comparisons, Pearson correlation analysis and multiple linear regression.

**Results:**

Parental self‐efficacy and family nutritional awareness differed significantly across selected sociodemographic and caregiving characteristics. Family support was the strongest predictor of parental self‐efficacy. Greater preparedness for disability, social security coverage, higher maternal education and cooperative child behaviour during dental examinations were also associated with higher self‐efficacy. Paternal education was not a significant predictor. Caregiving difficulty was positively associated with family nutritional awareness, suggesting that greater caregiving demands may encourage adaptive health information‐seeking and management behaviours. A high prevalence of dental pain was reported among children, indicating potential deficiencies in preventive oral health care, timely service access and caregiver education.

**Conclusion:**

Parental competence in managing the nutrition and oral health of children with disabilities is shaped by interpersonal, educational and structural resources. Interventions should strengthen family support, caregiver preparedness, access to social protection and practical guidance on preventive oral health and nutrition. Particular attention should be directed to families experiencing limited resources and children with unmet dental care needs in practice.

## Summary

- Family support was the strongest predictor of parental self‐efficacy among parents of children with disabilities.
- Preparedness for disability‐related caregiving, social security coverage, maternal education and children's cooperative behaviour during dental examinations was associated with higher parental self‐efficacy.
- Caregiving difficulty was positively associated with family nutrition and physical activity practices, suggesting that parents facing greater caregiving demands may become more attentive to health‐related caregiving practices.
- The high prevalence of parent‐reported dental pain among children with disabilities highlights the need for disability‐sensitive preventive oral health services and caregiver education.
- Integrated interventions addressing family support, caregiver preparedness, nutrition‐related practices and oral health may strengthen parental competence and improve care for children with disabilities.

## 1 Introduction

Children with disabilities experience multiple physical, psychological, nutritional and social health challenges that significantly affect their overall well‐being. These challenges may include metabolic disorders, nutritional deficiencies, poor oral hygiene, motor skill limitations, feeding difficulties and chronic health conditions (Çalışkan and Bayat 2016; Devnani and Hegde 2015; Karadağ and Bilsin 2016; Yılmaz et al. 2019). Feeding disorders are particularly common among children with special healthcare needs, with studies reporting that 30%–80% of these children experience feeding difficulties that may become chronic and lead to malnutrition, growth problems and developmental delays (Çoban and Özcebe 2019; Yoldaş and Yılmazer 2021). Inadequate nutrition may not only impair physical development but also exacerbate existing disability‐related health conditions (United Nations Children's Fund 2024). Consequently, early multidisciplinary interventions are considered essential for preventing long‐term adverse health outcomes and improving quality of life (Andrew and Sullivan 2010).

Oral and dental health represents another important yet frequently overlooked aspect of health among children with disabilities. Research consistently demonstrates that these children experience higher rates of untreated dental caries, poor oral hygiene, periodontal disease and unmet dental treatment needs than their typically developing peers (Mehta et al. 2024; Patidar et al. 2022). Recent disability‐focused evidence suggests that these oral health inequalities cannot be explained solely by individual oral hygiene practices. Rather, oral health outcomes are shaped by a complex interplay of family, social, behavioural and healthcare system factors. Al‐Mashhadani et al. (2024), in a systematic review and thematic synthesis, identified stigma, communication barriers, insufficient professional training, gaps in caregiver oral health education and inadequate medical–dental collaboration as major barriers to the utilization of dental services among children with disabilities. Similarly, Alwadi et al. (2024) emphasized the importance of caregiver education, service accessibility and disability‐sensitive care in improving access to oral healthcare. In addition, barriers to accessing dental services, insufficient caregiver oral health education and challenges in maintaining regular oral hygiene routines may contribute to poorer oral health outcomes in this population (Alwadi et al. 2024; Al‐Mashhadani et al. 2024; Krishnan et al. 2019). Parental‐perspective studies have also reported that families frequently encounter difficulties in finding appropriately trained dental professionals and face financial, transportation and service‐related barriers that restrict access to oral healthcare services (Asiri et al. 2024; Alwadi et al. 2024). Collectively, these findings indicate that oral health among children with disabilities should be examined within a broader family‐centred and systems‐oriented framework.

Because children with disabilities often depend heavily on caregivers for daily hygiene practices, dietary management and healthcare utilization, parental behaviours and beliefs become central determinants of health outcomes. One factor that has received increasing attention in family‐centred health research is parental self‐efficacy. According to Bandura's Social Cognitive Theory, self‐efficacy refers to individuals' beliefs in their capability to organize and execute actions required to manage specific situations successfully (Bandura 1997). The theory proposes that health‐related behaviours emerge through reciprocal interactions among personal factors, environmental influences and behavioural processes. Within this framework, parental self‐efficacy may influence children's oral and nutritional health through a knowledge–attitude–behaviour pathway. Parents with greater health‐related knowledge and stronger confidence are more likely to develop positive attitudes, supervise daily oral hygiene practices, seek preventive dental care and support healthier dietary habits.

Evidence from oral health research supports this theoretical mechanism. Parents with higher oral health self‐efficacy are more likely to establish regular toothbrushing routines, utilize preventive dental services and engage in behaviours that promote oral health (de Silva‐Sanigorski et al. 2013; Hamilton et al. 2018). More broadly, parents with stronger efficacy beliefs tend to adopt proactive health behaviours, adhere to treatment recommendations and maintain consistent caregiving routines (Coleman and Karraker 1998). Among families of children with disabilities, higher parental self‐efficacy has been associated with better coping strategies, greater involvement in care and improved adaptation to caregiving challenges (Jandrić and Kurtović 2021; Ronkainen et al. 2023). Educational attainment has also been identified as an important determinant of parental self‐efficacy because it may facilitate access to health information, problem‐solving skills and health‐related decision‐making (Davis‐Kean et al. 2021; Glatz et al. 2024).

Family support is another important component of Social Cognitive Theory, providing environmental resources that facilitate health‐promoting behaviours. Previous studies have shown that social support is associated with lower parenting stress, improved psychological well‐being and more positive family functioning (Hastings and Taunt 2002; Hosokawa and Katsura 2024). In families of children with disabilities or special healthcare needs, adequate support resources may help parents cope with caregiving demands and strengthen confidence in managing daily responsibilities (Cheng and Lai 2023). Parents who perceive stronger family and social support networks generally report higher levels of parental self‐efficacy (Fu et al. 2023; Zhang et al. 2024). In addition, social security coverage and economic stability may support parental confidence by reducing financial strain and improving access to healthcare services (Cheng and Lai 2023; Can et al. 2025). Recent evidence further suggests that parental self‐efficacy may mediate the relationship between social support and children's oral health behaviours, indicating that supportive family environments can indirectly improve oral health outcomes by strengthening parents' confidence in managing daily oral care routines (Shi et al. 2025).

Family nutrition awareness represents another important factor influencing health management among children with disabilities. Families with greater awareness of healthy nutrition practices may be better able to monitor dietary intake, prevent obesity or malnutrition and respond more effectively to feeding difficulties. This is particularly important because children with disabilities may experience feeding problems, selective eating, oral‐motor difficulties, gastrointestinal problems or dependence on caregivers for meal preparation and feeding support (Klein et al. 2023; Opoku et al. 2022). Therefore, nutrition‐related knowledge and family‐based dietary practices may play a protective role in promoting children's overall health and daily functioning.

Nutrition awareness is also closely related to oral health because frequent consumption of sugar‐rich foods, irregular eating habits and poor oral hygiene are well‐established risk factors for dental caries. Children with disabilities may be particularly vulnerable due to selective eating behaviours, sensory sensitivities, swallowing difficulties, medication use and dependence on caregivers for feeding and dietary management (Madaan and Sahu 2022). Consequently, nutrition awareness may represent an important behavioural pathway through which parental self‐efficacy influences oral health outcomes.

In addition to psychosocial and behavioural factors, sociodemographic characteristics may also influence parental self‐efficacy and health‐related caregiving behaviours. Variables such as educational level, social security status, preparedness for raising a child with a disability and perceived caregiving difficulty may affect parents' ability to manage daily health‐related responsibilities. Parents who are psychologically and practically prepared for raising a child with special needs often demonstrate stronger adaptive coping mechanisms and greater confidence in caregiving. Previous research has shown that parental adaptation is facilitated by effective coping resources and adequate support systems (Cheng and Lai 2023; Ronkainen et al. 2023). Similarly, caregiving burden and perceived caregiving difficulty may influence the sustainability of oral health and nutrition‐related routines, making these factors important contextual determinants within family‐centred care models.

Despite growing evidence regarding parental self‐efficacy, family support, nutrition‐related practices and oral health behaviours, these constructs have generally been examined separately in previous studies. Research integrating psychosocial, nutritional and oral health domains within a single conceptual framework remains limited, particularly among families of children with disabilities. This gap is important because oral health outcomes in this population are likely shaped by the combined influence of personal, familial, behavioural and healthcare‐system factors rather than any single determinant.

Grounded in Bandura's Social Cognitive Theory, the present study conceptualizes parental self‐efficacy as a central personal factor interacting with environmental support systems and behavioural mechanisms related to nutrition and oral health. Within this framework, family support represents an environmental resource, nutrition awareness functions as a behavioural regulation mechanism and oral health practices constitute key health‐related outcomes. The primary contribution of this study is that it simultaneously examines parental self‐efficacy, family support, nutrition awareness, caregiving difficulty and oral health practices within a single family‐centred analytical framework. By integrating these psychosocial, nutritional and behavioural domains, the study aims to provide a more comprehensive understanding of factors influencing oral health management among children with disabilities.

Therefore, the present study aims to determine the role of parental self‐efficacy and family support in the nutrition and oral health practices of children with disabilities and to examine how sociodemographic factors influence these relationships.

### 1.1 Aim of the Study

This study aimed to examine the interrelationships between parental self‐efficacy, family support, family nutrition awareness and oral‐dental health practices in parents of children with disabilities. In addition, the study investigated the influence of sociodemographic characteristics and caregiving‐related factors on parental self‐efficacy and family nutrition awareness.

### 1.2 Study Hypotheses

Based on the theoretical framework and existing literature, the following hypotheses were tested:

Hypothesis 1 *Parental self‐efficacy is positively associated with family nutrition awareness*.

Hypothesis 2 *Parental self‐efficacy is positively associated with favourable oral and dental hygiene practices of children with disabilities*.

Hypothesis 3 *Family support is positively associated with parental self‐efficacy*.

Hypothesis 4 *Family support is positively associated with family nutrition awareness*.

Hypothesis 5 *Higher parental education level is associated with higher parental self‐efficacy*.

Hypothesis 6 *Parents with social security demonstrate higher parental self‐efficacy compared to those without social security*.

Hypothesis 7 *Parents who were prepared for the birth of a child with disability report higher parental self‐efficacy than those who were not prepared*.

Hypothesis 8 *Perceived caregiving difficulty is positively associated with family nutrition awareness*.

Hypothesis 9 *Children*'*s cooperative behaviour during dental examination is positively associated with parental self‐efficacy*.

## 2 Methods

### 2.1 Study Design

This study employed a cross‐sectional, descriptive and correlational research design. The cross‐sectional nature of the study allows examination of associations between variables at a single point in time; however, causal inferences cannot be drawn from the findings due to the nonlongitudinal design.

### 2.2 Participants and Sampling

This cross‐sectional, descriptive and correlational study was conducted between January 2023 and June 2023 in multiple provincial centres across Turkey. The study population consisted of parents of children aged 0–18 years with diagnosed disabilities who were receiving educational, rehabilitation or healthcare services at the participating institutions.

Participants were recruited on a voluntary basis using a convenience sampling method. Random sampling was not performed. Parents who met the inclusion criteria were informed by the researchers about the study's purpose and procedures and invited to participate. The inclusion criteria were being the primary caregiver of a child with a disability, being 18 years of age or older and being able to communicate in Turkish. Parents who agreed to participate provided informed consent before data collection.

A total of 232 parents were registered at the Disability Services Unit during the data collection period. Of these, 208 met the inclusion criteria and were invited to participate. A total of 200 parents agreed to participate, corresponding to a participation rate of 96.2%. However, five participants were excluded due to incomplete data, resulting in a final analytic sample of 195 participants and an overall usable response rate of 93.8%. To better characterize the study population, information on children's disability types was collected based on parental reports of previously established medical diagnoses. Disability type information was available for 64 children. Among these valid responses, intellectual disability was the most frequently reported disability type (*n* = 42, 65.6%), followed by autism spectrum disorder (*n* = 8, 12.5%), physical disability (*n* = 7, 10.9%), hearing disability (*n* = 3, 4.7%), visual disability (*n* = 3, 4.7%) and multiple disabilities (*n* = 1, 1.6%).

Disability type was considered an important contextual characteristic because children with disabilities constitute a heterogeneous group with varying functional limitations, feeding difficulties, dependence on caregivers for oral hygiene and behavioural cooperation. However, because disability‐type information was available only for a subset of participants, these data were used descriptively and were not included as a primary comparative variable in the analysis. Diagnostic information was obtained solely from parental reports and was not independently verified through clinical assessment. Disability severity information was not available in the institutional records accessible to the research team and therefore could not be collected or examined as a study variable. Accordingly, the findings should be interpreted with consideration of potential heterogeneity in disability type, functional severity, caregiving experiences, nutritional management, behavioural cooperation and oral health outcomes among the participating children.

A nonprobability convenience sampling method was used. Parents were eligible to participate if they

1. had a child formally diagnosed with a disability,
2. were the primary caregiver,
3. were able to communicate in Turkish and
4. volunteered to participate.

Parents with severe communication difficulties or incomplete questionnaires were excluded from the analysis.

A total of 195 parents participated in the study. A post hoc power analysis conducted using G*Power 3.1 indicated that this sample size was sufficient to detect medium‐sized effects (*f* = 0.25) with 80% power at an alpha level of 0.05 in group comparison analyses. Therefore, the sample was considered statistically adequate for the planned inferential tests. For the multiple linear regression model, a post hoc sensitivity power analysis was performed based on the observed effect size. The final analytic sample comprised *N* = 195 participants, with no missing data in the regression variables. The observed model fit was *R* ^2^ = 0.171 with six predictors (*k* = 6). Accordingly, Cohen's effect size was calculated as *f* ^2^ = 0.206. Using *α* = 0.05, df1 = 6 and df2 = 188, the achieved statistical power for the overall model *F* test was 1 − *β* = 0.999, indicating very high sensitivity to detect the observed effect. However, because a nonprobability sampling strategy was employed, the findings should be generalized to the broader population of parents of children with disabilities in Turkey with caution.

### 2.3 Data Collection Procedure

Data were collected using a structured self‐report questionnaire administered to parents. Participants were approached during routine visits to the participating institutions providing services to children with disabilities and were informed by the researchers about the study objectives, procedures and voluntary nature of participation. Parents who agreed to participate provided written informed consent prior to data collection.

Questionnaires were completed either through face‐to‐face administration or under researcher supervision in a quiet setting at the participating institutions. Completing the questionnaire took approximately 25–30 min. Participation was entirely voluntary, and respondents were informed that they could withdraw from the study at any stage without any consequences.

Anonymity and confidentiality were strictly maintained throughout the study. No personally identifiable information was recorded. All data were securely stored and used solely for research purposes in accordance with the ethical approval obtained for the study. Of the parents approached, 15 either declined to participate or did not complete the questionnaire. Only fully completed questionnaires were included in the final analyses.

### 2.4 Study Variables and Measures

Data were collected using a structured questionnaire consisting of researcher‐developed forms and standardized scales. The questionnaire included sociodemographic characteristics, disability‐related variables, perceived preparedness for disability‐related caregiving, perceived caregiving difficulty, parental self‐efficacy, perceived family support, family nutrition and physical activity practices, and oral health‐related practices.

#### 2.4.1 Sociodemographic and Disability‐Related Variables

Sociodemographic data were collected using a researcher‐developed information form prepared based on previous literature (Çalışkan and Bayat 2016; Karadağ and Bilsin 2016; Masulani‐Mwale et al. 2016; Yılmaz et al. 2019). The form included questions on parental age, educational level, employment status, family structure, perceived income level, social security status, birth type and selected child‐ and disability‐related characteristics. Information regarding disability type was obtained through parental self‐report based on the child's previously established medical diagnosis. The research team did not independently verify diagnostic information through clinical assessment.

#### 2.4.2 Perceived Preparedness for Disability‐Related Caregiving

Perceived preparedness for disability‐related caregiving was assessed using a single self‐report item asking parents whether they would have felt prepared for raising a child with a disability if they had been informed about the condition before birth. Responses were categorized as *yes* or *no*. This variable was included as an indicator of parents' perceived psychological and practical readiness for caregiving responsibilities associated with disability.

#### 2.4.3 Perceived Caregiving Difficulty

Perceived caregiving difficulty was assessed using a single self‐report item asking parents whether they experienced difficulty caring for their child with a disability. Responses were categorized as *yes* or *no*. This variable was used to reflect parents' subjective perceptions of the burden and challenges associated with daily caregiving activities.

#### 2.4.4 Family and Child Oral‐Dental Health Data Form

Oral health‐related data were collected using a researcher‐developed Family and Child Oral‐Dental Health Data Form. This form assessed oral hygiene practices and oral health‐related experiences, including toothbrushing frequency and duration, brushing technique, dental visit history, fluoride use, toothache history and other oral health‐related behaviours. Items were structured as categorical variables for descriptive and comparative analyses.

#### 2.4.5 Family Nutrition and Physical Activity Scale (FNPA)

The FNPA, originally developed by Ihmels et al., evaluates family practices associated with childhood obesity risk. The Turkish adaptation was validated by Ekici et al. (2021). The scale consists of 20 items rated on a 4‐point Likert scale, with higher scores indicating healthier family nutrition and activity behaviours.

In the original validation study, Cronbach's alpha was reported as 0.724. In the present study, internal consistency was calculated as *α* = 0.66. Although this value indicates moderate reliability, it falls slightly below the commonly accepted threshold of 0.70. Therefore, results related to FNPA should be interpreted with caution, and this limitation is acknowledged in the discussion section.

#### 2.4.6 Parent Self‐Efficacy Scale (PSES)

The Parent Self‐Efficacy Scale consists of 17 items rated on a 7‐point Likert scale, measuring parents' perceived competence in caregiving tasks. The Turkish adaptation reported strong psychometric properties (Cavkaytar et al. 2014), with Cronbach's alpha of 0.95 and test–retest reliability of *r* = 0.79.

In the present study, internal consistency was found to be *α* = 0.98, indicating excellent reliability.

#### 2.4.7 Family Support Scale (FSS)

The Family Support Scale (Kaner 2003) measures perceived social support in parents of children with special educational needs. It includes 31 items across five subscales. Higher scores indicate greater perceived support.

In this study, Cronbach's alpha was calculated as *α* = 0.96, demonstrating excellent internal consistency.

### 2.5 Ethical Considerations

Before data collection, institutional permission was obtained from the relevant institution, and ethical approval was granted by the Kayseri University Ethics Committee (date: 2 December 2022; Decision No. 82). Meetings were conducted with the principals of all participating schools to explain the aim, scope and procedures of the study. Written informed consent was obtained from all participants through a consent form presented on the first page of the data collection instrument. The study was conducted in accordance with the principles of the Declaration of Helsinki, as revised in Brazil in 2013.

Written and verbal approvals of the participants were obtained with informed consent forms prepared according to the Declaration of Helsinki (as revised in Brazil 2013).

### 2.6 Statistical Analysis

Data were analysed using IBM SPSS Statistics Version 25.0. Descriptive statistics, including frequency, percentage, mean, standard deviation, minimum and maximum values, were calculated to summarize the data. Normality assumptions were evaluated using *Q*–*Q* plots and skewness and kurtosis coefficients. Values within ± 3 were considered acceptable for normal distribution. For group comparisons, independent samples *t* tests were used to compare two groups, whereas one‐way analysis of variance (ANOVA) was conducted for comparisons involving more than two groups. When significant differences were detected, Bonferroni post hoc tests were applied to identify pairwise differences. Effect sizes were calculated to determine the magnitude of group differences. Cohen's *d* was computed for *t* tests and interpreted as small (0.20), medium (0.50) and large (0.80). For ANOVA results, eta squared (*η* ^2^) values were calculated and interpreted as small (0.01), medium (0.06) and large (0.14). Pearson correlation analysis was performed to examine relationships between continuous variables. Correlation coefficients were interpreted as small (*r* = 0.10–0.29), medium (*r* = 0.30–0.49) and large (*r* ≥ 0.50). To identify predictors of parental self‐efficacy, a multiple linear regression analysis was conducted. Independent variables included mother's education level, father's education level, social security status, preparedness for disability, family support score and the child's cooperative behaviour during dental examination. Statistical significance was set at *p* < 0.05.

## 3 Results

This section reports the study findings.

A total of 195 parents participated in the study. Among the respondents, 75.4% were mothers, 17.9% were fathers and 6.7% were other caregivers. Regarding parental age, 52.8% of mothers were younger than 39 years, whereas 47.2% were 39 years or older. Among fathers, 60.0% were younger than 43 years, and 40.0% were 43 years or older.

In terms of family structure and educational characteristics, 86.7% of the children were living with both parents, and 79.0% of participants reported having a nuclear family structure. The most common educational level was primary school graduation for both mothers and fathers, reported by 37.4% of mothers and 33.3% of fathers. Only 13.3% of mothers reported being employed. Regarding perceived family income, 37.4% of participants stated that their income was lower than their expenses, 47.7% reported that their income was equal to their expenses and 14.9% reported that their income was higher than their expenses. In addition, 24.1% of participants reported having no social security coverage.

With respect to obstetric characteristics, 47.7% of children were delivered by caesarean section and 52.3% by vaginal delivery. Only 8.2% of participants reported that they had known before birth that their child would have a disability, whereas 91.8% had not known. Furthermore, 69.2% stated that they did not feel prepared for the birth of a child with a disability when they learned about the condition. Half of the participants, 50.3%, reported experiencing difficulties in caring for their child with a disability.

Disability type information was available for 64 children and was obtained through parental self‐report based on previously established medical diagnoses. Among children with available disability‐type data, intellectual disability was the most frequently reported diagnosis (65.6%), followed by autism spectrum disorder (12.5%) and physical disability (10.9%). Given that children with disabilities represent a heterogeneous population with varying functional limitations, feeding difficulties, dependence on caregivers for oral hygiene and behavioural characteristics, disability type was considered an important contextual characteristic when interpreting the findings (Table 1).

**TABLE 1:** Sociodemographic characteristics of participants (*N* = 195).

Variables	*n*	%
Respondent parent		
Mother	147	75.4
Father	35	17.9
Grandmother	13	6.7
Mother's age (mean ± SD = 39.46 ± 7.10)		
Under 39 years	103	52.8
39 years and above	92	47.2
Father's age (Mean ± SD = 42.86 ± 7.30)		
Under 43 years	117	60.0
43 years and above	78	40.0
Family status		
Parents together	169	86.7
Parents separated	25	12.8
Father deceased	1	0.5
Mother's education level		
Illiterate	11	5.6
Primary school graduate	73	37.4
Middle school graduate	26	13.3
High school graduate	45	23.1
University graduate	40	20.5
Father's education level		
Illiterate	8	4.1
Primary school graduate	65	33.3
Middle school graduate	29	14.9
High school graduate	52	26.7
University graduate	41	21.0
Mother's employment status		
Employed	26	13.3
Unemployed	169	86.7
Family income status		
Income less than expenses	73	37.4
Income equal to expenses	93	47.7
Income greater than expenses	29	14.9
Social security status		
Yes	148	75.9
No	47	24.1
Family type		
Nuclear family	154	79.0
Extended family	24	12.3
Fragmented family	17	8.7
Type of birth		
Normal	102	52.3
Caesarean	93	47.7
Knowing child would be born with disabilities		
Yes	16	8.2
No	179	91.8
Prepared for disability if informed		
Yes	60	30.8
No	135	69.2
Difficulty in caring for children with disabilities		
Yes	98	50.3
No	97	49.7
Child's disability type^a^		
Intellectual disability	42	65.6
Autism spectrum disorder	8	12.5
Physical disability	7	10.9
Hearing disability	3	4.7
Visual disability	3	4.7
Multiple disabilities	1	1.6
Total	195	100.0

^a^ Percentages for disability type are based on valid responses regarding disability type (*n* = 64). Disability type information was unavailable for the remaining participants.

**FIGURE 1:**
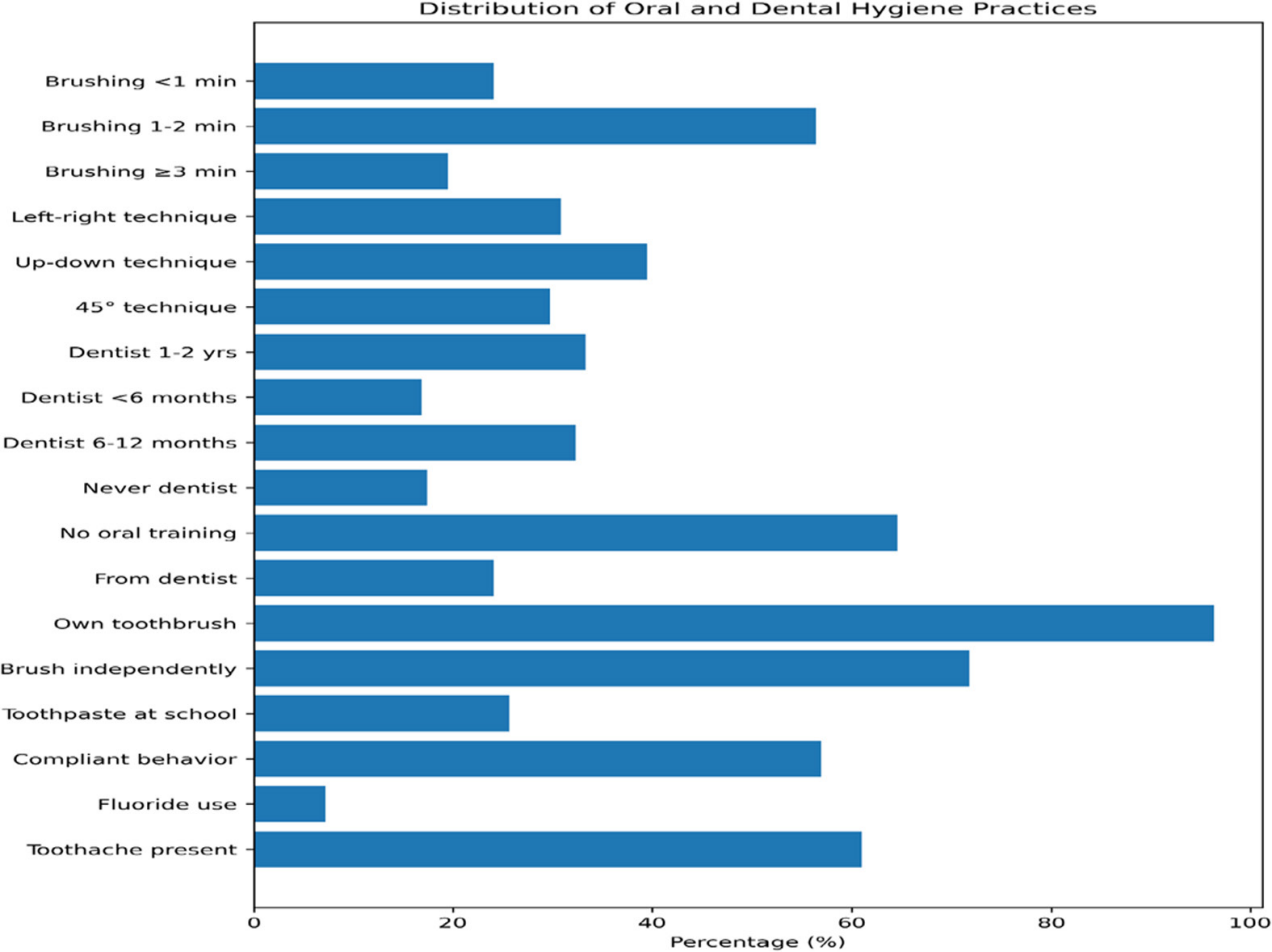
Distribution of oral and dental hygiene practices among children with disabilities. *Note:* Percentages represent the proportion of participants reporting each oral and dental hygiene practice. Most children owned a toothbrush (96.4%) and were able to brush independently (71.8%). However, a substantial proportion had not received any oral health education (64.6%), fluoride use was extremely low (7.2%) and 61.0% of the children currently experienced toothache. The correct brushing technique (45° angle) was reported by only 29.7% of participants.

The distribution of oral and dental hygiene practices among parents and children with disabilities is presented in Figure 1.

Regarding brushing duration, 24.1% of children brushed for less than 1 min, 56.4% brushed for 1–2 min and 19.5% brushed for 3 min or longer. In terms of brushing technique, 30.8% used a left–right motion, 39.5% used an up‐down motion and only 29.7% reported using the recommended 45° angle technique.

Concerning dental visits, 33.3% of participants reported visiting a dentist within the past 1–2 years, 32.3% within 6–12 months, 16.9% within the past 6 months, and 17.4% had never visited a dentist.

Overall, 64.6% of participants had not received oral health education; among those who had, 24.1% reported receiving education from a dentist. Among the children included in the study, 25.6% had toothpaste available at school, and 7.2% used fluoride tablets. Additionally, 56.9% of the children demonstrated cooperative behaviour during dental examinations (see Figure 1).

According to Table 2, Family Nutrition Awareness Scale scores differed significantly according to type of birth and preparedness for disability if informed before birth (*p* < 0.05). Participants who reported being prepared for disability and those whose children were born through normal vaginal delivery had higher nutrition awareness scores. No significant differences were found according to respondent parent, parental age, parental education level, family income status or family type (*p* > 0.05).

**TABLE 2:** Family Nutrition Awareness Questionnaire scores according to sociodemographic characteristics of parents.

Variables		Min	Max	$\bar{X}$	SD	Test value	*p*	Bonferroni
Parent respondent	Mother	29.00	56.00	43.31	5.03	1.755***	0.176	
	Father	33.00	56.00	43.83	6.01			
	Other	40.00	53.00	46.08	4.09			
Maternal age status	Under 39 years old	31.00	56.00	43.81	5.18	0.303**	0.539	
	39 years and older	29.00	56.00	43.35	5.21			
Father's age status	Under 43 years old	29.00	56.00	90.51	29.23	2.500**	0.955	
	43 years and older	33.00	56.00	91.37	27.03			
Mother's education status	Illiterate (1)	36.00	55.00	45.36	5.61	1.329***	0.261	
	Primary school graduate (2)	33.00	56.00	44.38	5.34			
	Secondary school graduate (3)	29.00	54.00	43.31	4.93			
	High school graduate (4)	34.00	53.00	42.89	4.82			
	University graduate (5)	31.00	54.00	43.59	5.19			
Father's education status	Illiterate (1)	36.00	54.00	45.75	6.18	1.459***	0.216	
	Primary school graduate (2)	33.00	56.00	44.28	5.33			
	Secondary school graduate (3)	29.00	49.00	42.58	4.04			
	High school graduate (4)	34.00	53.00	43.87	5.32			
	University graduate (5)	31.00	54.00	42.44	5.18			
Family income status	Income less than expenses	29.00	55.00	44.01	5.69	0.768***	0.465	
	Income more than expenses	34.00	54.00	44.07	4.29			
	Income equals expenses	31.00	56.00	43.11	5.03			
Family type	Nuclear family	29.00	56.00	43.34	5.08	1.982***	0.141	
	Extended family	35.00	54.00	45.54	6.02			
	Fragmented family	34.00	56.00	43.06	5.19			
Type of birth	Caesarean	31.00	56.00	42.21	5.12	−3.84**	< 0.001*	
	Normal	29.00	55.00	44.97	4.90			
Prepared for disability if informed	Yes	34.00	56.00	45.07	5.12	2.70**	0.008*	
	No	29.00	56.00	42.93	5.11			

* *p* < 0.05.

** Independent *t* test.

*** One‐way analysis of variance.

Table 3 shows that Parental Self‐Efficacy Scale scores differed significantly according to maternal educational level (*p* = 0.038). Post hoc Bonferroni analyses indicated that participants whose mothers had a university education reported significantly higher parental self‐efficacy scores than those whose mothers were illiterate or had completed primary school (5 > 1, 5 > 2).

**TABLE 3:** Parental Self‐Efficacy Scale scores according to sociodemographic characteristics of parents.

Variables		Min	Max	$\bar{X}$	SD	Test value	*p*	Bonferroni
Parent respondent	Mother	17.00	119.00	91.94	28.61	0.510***	0.601	
	Father	17.00	119.00	86.57	30.46			
	Other	64.00	116.00	90.15	17.23			
Maternal age status	Under 39 years old	17.00	119.00	92.40	27.01	0.804**	0.422	
	39 years and older	17.00	119.00	89.13	29.74			
Father's age status	Under 43 years old	17.00	119.00	90.51	29.23	−0.207**	0.836	
	43 years and older	17.00	119.00	91.37	27.03			
Mother's education status	Illiterate (1)	25.00	112.00	76.64	33.01	2.585***	0.038*	5 > 1, 5 > 2
	Primary school graduate (2)	17.00	119.00	86.68	30.46			
	Secondary school graduate (3)	17.00	119.00	96.19	26.10			
	High school graduate (4)	17.00	119.00	89.47	30.16			
	University graduate (5)	34.00	119.00	100.48	18.02			
Father's education status	Illiterate (1)	25.00	112.00	87.00	30.45	2.511***	0.043*	3 > 2, 5 > 2
	Primary school graduate (2)	17.00	119.00	83.00	31.55			
	Secondary school graduate (3)	17.00	119.00	97.28	24.80			
	High school graduate (4)	17.00	119.00	91.60	28.82			
	University graduate (5)	27.00	119.00	98.59	21.12			
Mother's employment status	Yes	17.00	119.00	90.85	27.27	−0.002**	0.998	
	No	17.00	119.00	90.86	28.54			
Family income status	Income less than expenses	17.00	119.00	89.18	29.25	0.710***	0.493	
	Income more than expenses	35.00	119.00	96.48	26.18			
	Income equals expenses	17.00	119.00	90.42	28.26			
Social security status	Yes	17.00	119.00	93.22	26.42	2.084**	0.039*	
	No	17.00	119.00	83.43	32.76			
Family type	Nuclear family	17.00	119.00	89.90	28.55	0.442***	0.643	
	Extended family	25.00	119.00	93.67	29.34			
	Fragmented family	17.00	119.00	95.59	25.23			
Type of birth	Caesarean	17.00	119.00	93.35	29.74	1.178**	0.240	
	Normal	25.00	119.00	88.58	26.87			
Knowing child would be born with disabilities	Yes	48.00	119.00	97.69	20.50	1.008**	0.315	
	No	17.00	119.00	90.25	28.86			
Prepared for disability if informed	Yes	33.00	119.00	99.25	18.25	2.809**	0.005*	
	No	17.00	119.00	87.13	31.11			

* *p* < 0.05.

** Independent *t* test.

*** One‐way analysis of variance.

Parental self‐efficacy scores also differed significantly according to paternal educational level (*p* = 0.043). Bonferroni comparisons revealed that participants whose fathers had completed middle school or university education reported higher parental self‐efficacy scores than those whose fathers had completed primary school (3 > 2, 5 > 2).

A statistically significant difference was also observed according to social security status (*p* = 0.039). Participants with social security reported higher parental self‐efficacy scores than those without social security.

Finally, parental self‐efficacy scores differed significantly according to preparedness for disability if informed before birth (*p* = 0.005). Participants who reported that they would have been prepared for raising a child with a disability if informed before birth had significantly higher parental self‐efficacy scores than those who reported that they would not have been prepared.

No statistically significant differences were found according to respondent parent, parental age, maternal employment status, family income, family type, type of birth or prior knowledge that the child would be born with a disability (*p* > 0.05) (Table 3).

As a result, it is observed that there is a statistically significant difference in parental self‐efficacy scale scores based on the child's compliant behaviour during the examination (*p* < 0.05). It has been determined that the parental self‐efficacy scale scores of participants whose children exhibit compliant behaviour during the examination are higher than those of participants whose children do not exhibit such behaviour. There is no statistically significant difference in Family Support Scale scores according to the oral and dental hygiene practices of parents and children with disabilities (*p* > 0.05) (Table 4).

**TABLE 4:** Mean scores of Parent Self‐Efficacy and Family Support Scale according to oral and dental hygiene practices of parents and children with disabilities.

Variables		Parental Self‐Efficacy Scale						Family Support Scale					
		Min	Max	$\bar{X}$	SD	Test value	*p*	Min	Max	$\bar{X}$	SD	Test value	*p*
Tooth brushing duration	Less than 1 min	17.00	119.00	89.66	28.25	2.039***	0.133	31.00	93.00	71.19	16.35	2.315***	0.102
	1–2 min	17.00	119.00	93.95	26.29			34.00	93.00	75.12	16.06		
	3 min or more	17.00	119.00	83.39	32.93			31.00	93.00	69.00	17.75		
Tooth brushing technique	Left to right	25.00	119.00	85.93	30.24	1.345***	0.263	31.00	93.00	70.93	17.18	0.757***	0.470
	Up and down	17.00	119.00	93.56	25.83			31.00	93.00	73.34	15.51		
	45° angle (correct method)	17.00	119.00	92.36	29.20			34.00	93.00	74.62	17.37		
Last visit to the dentist	Within 1–2 years	29.00	119.00	94.92	25.55	0.723***	0.540	31.00	93.00	74.00	17.44	0.784***	0.504
	Within 6 months	17.00	119.00	90.18	26.72			31.00	93.00	74.42	15.81		
	Within 6 months to 1 year	17.00	119.00	88.89	30.26			38.00	93.00	73.25	15.73		
	Never	17.00	119.00	87.38	31.27			33.00	93.00	69.12	17.29		
The child has his/her own toothbrush	Yes	17.00	119.00	91.34	27.88	1.239**	0.217	31.00	93.00	73.22	16.45	1.065**	0.288
	No	27.00	119.00	77.86	38.45			38.00	93.00	66.43	19.91		
Child's ability to brush his/her own teeth	Yes	17.00	119.00	92.44	27.99	1.509***	0.224	31.00	93.00	73.34	16.64	0.156***	0.856
	No	17.00	119.00	85.38	29.95			31.00	93.00	71.86	17.01		
	Sometimes	95.00	111.00	101.40	5.94			63.00	93.00	74.00	11.66		
Availability of toothpaste at school	Yes	17.00	119.00	89.70	27.00	−0.334**	0.739	31.00	93.00	70.08	17.79	−1.438**	0.152
	No	17.00	119.00	91.26	28.82			31.00	93.00	73.98	16.08		
The child shows compliant behaviour during the examination	Yes	25.00	119.00	94.53	27.04	2.103**	0.037*	31.00	93.00	74.52	16.47	1.499**	0.135
	No	17.00	119.00	86.00	29.35			31.00	93.00	70.94	16.60		
Fluorine tablet usage status	Yes	17.00	116.00	91.29	36.22	0.059**	0.953	45.00	93.00	70.29	16.74	−0.630**	0.529
	No	17.00	119.00	90.82	27.73			31.00	93.00	73.19	16.59		
Toothache in a child	Yes	17.00	119.00	91.31	26.77	0.050***	0.952	31.00	93.00	71.70	16.55	0.946***	0.390
	No	17.00	119.00	89.97	30.37			34.00	93.00	74.81	16.86		
	Sometimes	17.00	119.00	91.44	35.26			48.00	93.00	76.33	14.51		

* *p* < 0.05.

** Independent *t* test.

*** One‐way analysis of variance.

Pearson correlation analysis revealed several statistically significant associations among the study variables. A positive correlation was found between parental self‐efficacy and family support (*r* = 0.291, *p* < 0.001), indicating that higher perceived family support was associated with higher parental self‐efficacy. Family nutrition awareness was positively correlated with family support (*r* = 0.177, *p* = 0.013) and caregiving difficulties (*r* = 0.267, *p* < 0.001). No statistically significant correlation was observed between family nutrition awareness and parental self‐efficacy (*r* = 0.133, *p* = 0.064), between parental self‐efficacy and caregiving difficulties (*r* = 0.068, *p* = 0.343) or between family support and caregiving difficulties (r = 0.132, *p* = 0.066). Although several statistically significant associations were identified, the correlation coefficients were generally small, suggesting modest relationships among the variables (Table 5).

**TABLE 5:** The relationship between family nutrition awareness and parental self‐efficacy, family support and care difficulties for children with disabilities.

Scales		1	2	3	4
1‐ Family Nutrition Awareness Survey Aile	*r*	1.000	0.133	0.177	0.267
	*p*	—	0.064	0.013*	0.000**
2‐ Parental Self‐Efficacy Scale	*r*		1.000	0.291	0.068
	*p*		—	0.000**	0.343
3‐ Family Support Scale	*r*			1.000	0.132
	*p*			—	0.066
4‐ Difficulties in the care of a children with disabilities	*r*				1.000
	*p*				—

* *p* < 0.05.

** *p* < 0.001.

### 3.1 Model Statistics

**Table:** 

Statistic	Value
*R* ^2^	0.171
Adjusted *R* ^2^	0.144
*F*(6, 188)	6.45
*p*	< 0.001
Cohen's *f* ^2^	0.206 (moderate effect)

### 3.2 Assumption Diagnostics

All tolerance values were above 0.70, and VIF values were below 2.00, indicating no multicollinearity concerns.

A multiple linear regression analysis was conducted to identify factors associated with parental self‐efficacy among parents of children with disabilities (*N* = 195). The overall model was statistically significant, *F*(6, 188) = 6.45, *p* < 0.001, explaining approximately 17% of the variance in parental self‐efficacy (*R* ^2^ = 0.171; adjusted *R* ^2^ = 0.144). The calculated effect size (Cohen's *f* ^2^ = 0.206) indicated a moderate level of practical significance. Examination of multicollinearity diagnostics revealed tolerance values above 0.70 and VIF values below 2.00, indicating no multicollinearity concerns.

Among the predictors, family support emerged as the strongest predictor of parental self‐efficacy (*β* = 0.29, *p* < 0.001). Higher levels of perceived family support were associated with higher parental self‐efficacy scores.

Preparedness for disability was also a significant positive predictor (*β* = 0.18, *p* = 0.006). Parents who reported that they would have been prepared for raising a child with a disability if informed before birth reported higher parental self‐efficacy scores.

Social security status significantly predicted parental self‐efficacy (*β* = 0.14, *p* = 0.033). Parents with social security coverage reported higher parental self‐efficacy scores than those without social security coverage.

Maternal education level was a significant predictor (*β* = 0.16, *p* = 0.007), with higher educational attainment associated with higher parental self‐efficacy. In contrast, paternal education level was not a significant predictor (*β* = 0.09, *p* = 0.130).

Finally, child cooperative behaviour significantly predicted parental self‐efficacy (*β* = 0.13, *p* = 0.035). Parents of children who demonstrated cooperative behaviour reported higher parental self‐efficacy scores.

Overall, these findings suggest that parental self‐efficacy is associated with family support, preparedness for disability, social security status, maternal educational level and child cooperative behaviour (Table 6).

**TABLE 6:** Multiple linear regression analysis predicting parental self‐efficacy (*N* = 195).

Predictor variable	*B*	SE	*β*	*t*	*p*	95% CI for *B*	Tolerance	VIF
Family support	0.42	0.09	0.29	4.61	< 0.001	[0.24, 0.60]	0.81	1.23
Preparedness for disability	5.87	2.10	0.18	2.79	0.006	[1.73, 10.01]	0.88	1.14
Social security status	6.12	2.85	0.14	2.15	0.033	[0.50, 11.74]	0.84	1.19
Maternal education level	3.95	1.45	0.16	2.72	0.007	[1.09, 6.81]	0.76	1.31
Paternal education level	2.10	1.38	0.09	1.52	0.130	[−0.62, 4.82]	0.79	1.26
Child's cooperative behaviour	4.36	2.04	0.13	2.13	0.035	[0.33, 8.39]	0.86	1.16

Abbreviations: *β*, standardized regression coefficient; CI, confidence interval.

## 4 Discussion

This study examined the interrelationships among parental self‐efficacy, family support, family nutrition awareness, caregiving difficulties and oral health outcomes in families raising children with disabilities. Grounded in Social Cognitive Theory (Bandura 1997), the findings indicate that parental self‐efficacy is shaped primarily by environmental and structural determinants rather than caregiving burden alone. Concurrently, the results reveal substantial unmet needs in oral and nutritional health management among children with disabilities.

### 4.1 Parental Self‐Efficacy as a Contextually Embedded Construct (Hypotheses 3, 5–7 and 9)

One of the most important findings of the study was that family support emerged as the strongest predictor of parental self‐efficacy, supporting Hypothesis 3. Family support was significantly associated with parental self‐efficacy in both correlational and regression analyses. This finding is consistent with previous research, indicating that perceived social support enhances parenting competence and buffers caregiving stress among families of children with disabilities (Hosokawa and Katsura 2024; Fu et al. 2023; Zhang et al. 2024). From the perspective of Social Cognitive Theory, supportive family environments may strengthen efficacy beliefs through social persuasion, emotional reassurance and practical assistance (Bandura 1997). The persistence of this relationship after controlling for other variables highlights the central role of family support in shaping parental confidence.

Socioeconomic resources also appeared to play an important role in parental self‐efficacy. Consistent with Hypothesis 6, parents with social security coverage reported significantly higher self‐efficacy levels. Access to healthcare services and financial security may reduce uncertainty and caregiving‐related stress, thereby strengthening parents' confidence in managing their child's needs (Cheng and Lai 2023). This finding suggests that parental self‐efficacy should be understood not only as an individual psychological construct but also as a reflection of broader social and structural conditions.

Preparedness for raising a child with a disability was another significant determinant of parental self‐efficacy, supporting Hypothesis 7. Parents who reported being prepared for disability demonstrated higher self‐efficacy levels than those who were not prepared. Access to information, anticipatory guidance and adaptive coping resources may facilitate parental adjustment and strengthen confidence during caregiving processes (Ronkainen et al. 2023; Cheng and Lai 2023). Previous research has similarly emphasized that social support and preparedness may help parents adapt more effectively to disability‐related challenges (Fu et al. 2023).

Educational level was also associated with parental self‐efficacy, partially supporting Hypothesis 5. Maternal education emerged as a significant predictor, whereas paternal education did not. Mothers with higher educational attainment may possess greater access to health information, stronger communication skills and greater capacity to navigate healthcare systems and support services (Davis‐Kean et al. 2021; Glatz et al. 2024). Previous studies have similarly reported stronger parenting competence and self‐efficacy among more educated parents of children with developmental disabilities (Agusthia et al. 2021; Jandrić and Kurtović 2021; Laçin and Doğan 2024).

In contrast, paternal education was not a significant predictor of parental self‐efficacy. This finding should be interpreted cautiously and should not be viewed as evidence that fathers' educational resources are unimportant. Rather, it may reflect differences in caregiving involvement and respondent composition. Because mothers constituted the majority of respondents and often assume primary caregiving responsibilities, maternal educational resources may have a more direct influence on caregiving confidence and health‐management behaviours (Sharabi and Marom‐Golan 2018). Future research should further explore the distinct contributions of maternal and paternal roles in families of children with disabilities.

Finally, children's cooperative behaviour during dental examinations was significantly associated with parental self‐efficacy, supporting Hypothesis 9. This finding is consistent with the principle of reciprocal determinism proposed by Social Cognitive Theory, which emphasizes bidirectional interactions between personal beliefs and behavioural outcomes (Bandura 1997). Parents with stronger efficacy beliefs may establish routines that facilitate cooperation, whereas positive child behaviours may reinforce parents' confidence in their caregiving abilities.

### 4.2 Parental Self‐Efficacy and Oral Health Practices (Hypothesis 2)

The findings provided only partial support for Hypothesis 2. Although parental self‐efficacy was not significantly associated with most oral health practices, parents whose children demonstrated cooperative behaviour during dental examinations reported significantly higher self‐efficacy levels. This result suggests that parental self‐efficacy may be more closely related to behavioural and interactional aspects of oral healthcare than to routine oral hygiene practices alone. Oral health outcomes among children with disabilities are influenced by multiple factors, including disability‐related functional limitations, behavioural challenges, access to dental services, socioeconomic conditions and caregiver burden. Therefore, parental self‐efficacy should be considered one component within a broader ecological context influencing oral healthcare behaviours.

One of the most notable findings of the present study was the high prevalence of parent‐reported toothache among children with disabilities, with 61.0% of children reported to have experienced toothache. This finding may indicate substantial unmet oral health needs within this population and is consistent with previous evidence showing that children with disabilities are at increased risk of poor oral health outcomes, including dental caries, poor oral hygiene and unmet dental treatment needs (Patidar et al. 2022; Madaan and Sahu 2022).

However, given the cross‐sectional design of the study and the reliance on parental reports, the underlying causes of toothache cannot be determined. Toothache may be influenced by multiple interrelated factors, including dietary habits, socioeconomic conditions, irregular dental attendance, oral hygiene practices, behavioural management difficulties and disability‐related functional limitations. Therefore, this finding should be interpreted cautiously and should not be considered evidence of a causal relationship or a definitive indicator of deficiencies in preventive dental services.

Importantly, the high prevalence of toothache coexisted with high rates of toothbrush ownership (96.4%) and independent toothbrushing (71.8%). This observation suggests that access to oral hygiene tools alone may not be sufficient to ensure effective preventive oral care. In the present study, only 29.7% of participants reported correct toothbrushing technique, and 64.6% of parents had not received formal oral health education. These findings highlight the importance of structured oral health education, caregiver guidance and professional support for families of children with disabilities.

Recent disability‐focused studies have further demonstrated that oral health inequalities among children with disabilities are shaped by multiple barriers operating at the family, community and healthcare‐system levels. These barriers include stigma, communication difficulties, inadequate caregiver education, insufficient professional training and limited access to disability‐sensitive dental services (Al‐Mashhadani et al. 2024; Alwadi et al. 2024; Asiri et al. 2024). Together, these findings underscore the importance of family‐centred and system‐oriented approaches to oral healthcare promotion.

Toothache and untreated oral health problems may also have broader implications for children with disabilities, including potential effects on feeding, nutrition, comfort, daily functioning and quality of life. Feeding difficulties are already common among children with disabilities, and oral pain may further complicate eating behaviours and nutritional management (Çoban and Özcebe 2019; United Nations Children's Fund 2024; Yoldaş and Yılmazer 2021). Consequently, improving oral health among children with disabilities may require not only preventive dental services but also integrated interventions combining caregiver education, nutritional counselling, psychosocial support and accessible disability‐sensitive healthcare services.

### 4.3 Nutrition Awareness, Family Support, and Caregiving Demands (Hypotheses 1, 4 and 8)

The findings revealed a complex pattern regarding nutrition awareness. Contrary to Hypothesis 1, parental self‐efficacy was not significantly associated with family nutrition awareness. This suggests that caregiving confidence and nutrition‐related knowledge represent related but distinct constructs. Although self‐efficacy reflects perceived competence in managing caregiving responsibilities, nutrition awareness may depend more heavily on access to information, educational opportunities and exposure to health promotion resources.

In contrast, family support demonstrated a modest but significant positive association with nutrition awareness, supporting Hypothesis 4. Social support networks may facilitate information sharing, reinforce healthy family routines and enhance parents' ability to implement nutrition‐related recommendations (Delaney et al. 2022). However, the relatively small effect size indicates that family support alone may not be sufficient to improve nutrition awareness without structured educational interventions and professional guidance.

Consistent with Hypothesis 8, caregiving difficulty was positively associated with nutrition awareness. Parents who reported greater caregiving challenges also reported higher levels of nutrition‐related awareness and family health practices. Rather than indicating that caregiving difficulties reduce awareness, this finding may reflect adaptive coping processes and increased information seeking. Previous studies have emphasized that families caring for children with disabilities frequently encounter feeding difficulties and nutrition‐related concerns that require active management and continuous learning (Opoku et al. 2022; Klein et al. 2023).

Nevertheless, because of the study's cross‐sectional design, the direction of this relationship cannot be determined. It is possible that increased caregiving challenges promote information seeking, that greater awareness increases recognition of caregiving difficulties or that both are influenced by other factors such as disability severity, feeding problems, socioeconomic conditions or access to professional support.

Taken together, the findings support the view that parental self‐efficacy, family support, nutrition awareness and oral health‐related factors are interconnected but distinct components of family health management. Consistent with Social Cognitive Theory, health‐related outcomes among children with disabilities appear to emerge from dynamic interactions among personal beliefs, family resources, caregiving experiences and environmental conditions. These findings underscore the importance of family‐centred interventions that combine oral health education, nutritional counselling, psychosocial support and accessible healthcare services to improve both child and family outcomes.

### 4.4 Clinical and Practical Implications

The present findings also have important implications for clinical practice and family‐centred interventions. Families with limited financial resources may experience greater difficulties in accessing preventive and specialized dental services for children with disabilities (Al‐Mashhadani et al. 2024; Asiri et al. 2024). Healthcare professionals play a critical role in supporting families through oral health education, counselling and coordinated care pathways. Previous research has emphasized the importance of disability‐sensitive services and stronger collaboration between families and healthcare providers in improving oral healthcare utilization (Alwadi et al. 2024). Furthermore, parent‐focused oral health education programmes may strengthen caregivers' confidence in managing daily oral hygiene practices and promote positive oral health behaviours. Family‐centred oral health promotion programmes have been shown to improve parental engagement and facilitate the establishment of preventive oral health routines within the home environment. Recent intervention studies have also demonstrated that caregiver‐targeted educational approaches can improve oral hygiene practices and support better oral health outcomes among children with special needs. Therefore, family‐centred oral health promotion programmes that combine caregiver education, individualized counselling and ongoing professional support may represent an effective strategy for improving oral health outcomes among children with disabilities (Yu et al. 2022).

### 4.5 Disability‐Specific Considerations

The disability profile of the study population should be considered when interpreting the findings. Among participants who reported disability type, intellectual disability represented the largest diagnostic category, followed by autism spectrum disorder and physical disabilities. Differences in cognitive functioning, motor abilities, feeding challenges, behavioural cooperation and dependence on caregivers may influence both oral health behaviours and caregiving experiences. Consequently, the observed relationships may not be equally applicable across all disability groups. Future studies should examine these associations within specific disability categories and levels of functional impairment.

### 4.6 Integrated Interpretation and Implications

Collectively, the findings suggest that parental self‐efficacy is associated with family support, socioeconomic security, preparedness for disability, maternal educational level and child cooperative behaviour, whereas nutritional awareness appears to be more closely associated with caregiving demands and informational resources. Furthermore, the high prevalence of parent‐reported toothache may indicate unmet oral health needs that extend beyond individual‐level behaviours and involve broader family and healthcare‐system factors.

Families raising children with disabilities require structured, evidence‐based guidance to address complex nutritional and oral health needs. Inadequate nutritional management and poor oral health practices contribute to compounded health risks in this population (Klein et al. 2023; Patidar et al. 2022). Educational attainment enhances caregiving competence (Jandrić and Kurtović 2021), social support facilitates effective use of health information (Delaney et al. 2022), early preparation strengthens parental adaptation (Ronkainen et al. 2023) and socioeconomic security enhances healthcare access (Can et al. 2025). Professional oral health education delivered by dental practitioners significantly improves preventive behaviours (Mueller et al. 2022) and aligns with ADA guidelines (ADA 2024).

These findings underscore the necessity of comprehensive, family‐centred and multidisciplinary interventions integrating oral health education, nutritional counselling, psychosocial support and structural access improvements. Consistent with disability health frameworks (Turan Gürhopur and İşler Dalgıç 2017), multilevel strategies are essential to improve both parental well‐being and child health outcomes.

### 4.7 Strengths and Limitations

This study contributes to the literature by integrating parental self‐efficacy, family support, nutrition awareness, caregiving difficulty and oral health outcomes within a single analytical framework. The use of regression analysis strengthened the identification of independent predictors of parental self‐efficacy.

However, several limitations should be considered. First, the cross‐sectional design prevents causal interpretation of the relationships among study variables. Second, data were based on parental self‐reports, which may have introduced recall bias, response bias and subjective interpretation. In particular, oral health outcomes, including toothache history and oral hygiene practices, were not verified through clinical dental examinations. Third, disability type information was available only for a subset of participants and was based on parental report rather than independent clinical verification. Disability severity and functional impairment levels were not assessed. Because oral health needs, feeding difficulties, caregiving burden and behavioural cooperation may differ according to disability type and severity, the findings should be interpreted with caution and may not fully reflect differences across diagnostic or functional groups. Fourth, the Family Nutrition and Physical Activity Scale demonstrated moderate internal consistency (*α* = 0.66), and findings related to this measure should therefore be interpreted cautiously. Finally, the use of convenience sampling limits the generalizability of the findings.

Future longitudinal and intervention‐based studies incorporating clinical oral health assessments are needed to clarify causal pathways and determine whether structured family‐centred oral health and nutrition programmes improve outcomes among children with disabilities.

## 5 Conclusions

This study showed that parental self‐efficacy among families of children with disabilities was associated with educational level, social security status, preparedness for disability and family support. Family support appeared to be an important factor related to parental confidence, emphasizing the role of social and environmental resources in caregiving processes. Although caregiving difficulty was associated with greater nutritional awareness, parental self‐efficacy and nutrition awareness appeared to represent related but distinct constructs.

The high prevalence of parent‐reported dental pain may indicate unmet oral health needs in this population. However, given the cross‐sectional and self‐report nature of the study, this finding should be interpreted cautiously and cannot be considered direct evidence of systemic deficiencies in preventive dental services.

Overall, the findings support the need for family‐centred interventions that integrate oral health education, nutritional counselling, psychosocial support and improved access to professional guidance. Programmes that strengthen both parental empowerment and service accessibility may contribute to better health outcomes for children with disabilities and their families.

## Author Contributions

**Serdal Deniz:** conceptualization, supervision, resources, writing – review and editing, methodology, writing – original draft, software, validation, visualization, data curation. **Derya Evgin:** writing – review and editing, methodology, validation, formal analysis, project administration, resources, funding acquisition. **Rıdvan Karabulut:** supervision, investigation, methodology, writing – original draft.

## Funding

The authors have nothing to report.

## Ethics Statement

Before the study, institutional permission was obtained from the institution, whereas ethical approval was obtained from the university ethics committee (date: 2 December 2022, No: 82). Meetings were held with school principals of all the schools, and the research aim, content and method were explained to them. Participants' consent for the study was obtained on the first page of the data collection tool.

## Conflicts of Interest

The authors declare no conflicts of interest.

## Data Availability

The datasets generated and/or analysed during the current study are not publicly available but are available from the corresponding author on reasonable request.
